# Impact of Neck and Shoulder Pain on Health-Related Quality of Life in Adults in Saudi Arabia

**DOI:** 10.7759/cureus.59252

**Published:** 2024-04-29

**Authors:** Majdi Hashem, Reem A Almohaini, Turki Melfi Alharbi, Muhamad Muslim Aljurfi, Saad Abdullah Alzmamy, Fahad Sulaiman Alhussainan, Abdulmalik Essa Aldhafyan

**Affiliations:** 1 Surgery, Faculty of Medicine, Imam Mohammad Ibn Saud Islamic University, Riyadh, SAU; 2 Orthopedic Surgery, King Fahd University Hospital, Al Khobar, SAU; 3 College of Medicine, Imam Mohammad Ibn Saud Islamic University, Riyadh, SAU; 4 Urology, Security Forces Hospital, Riyadh, SAU

**Keywords:** shoulders, saudi arabia, quality of life, pain, neck, musculoskeletal

## Abstract

Background: Musculoskeletal pain is widely recognized as a prevalent public health issue that affects individuals of various genders and age groups. This study aimed to assess the influence of neck and shoulder pain on the quality of life (QoL) of adult individuals living in Saudi Arabia.

Method: This is a cross-sectional study using an online-administered questionnaire that was distributed via online platforms in Saudi Arabia for the duration between January and June 2023. The 36-Item Short Form Health Survey (SF-36) questionnaire was used to estimate participants QoL. Binary logistic regression analysis was used to identify predictors of better QoL.

Results: A total of 6601 participants were involved in this study. The majority of the participants (76.8%, n = 4610) reported that they had muscle tension, stiffness, pressure, or dull pain in the neck and shoulder area. The mean pain score for the study participants was 4.0 (SD: 2.9), which indicates mild degree of pain. When the participants were asked about their health today, the mean score was 5.9 (SD: 3.9), which indicates moderate health status. The mean SF-36 score for the study participants was 58.16 (17.4), which demonstrates moderate quality of life. Binary logistic regression analysis showed that male gender, younger age, being married or divorced, and having lower BMI (less than 25.3 kg/cm^2^) were predictors of better QoL (p < 0.05).

Conclusion: Notwithstanding the participants' moderate evaluations of their current health and quality of life, certain demographic attributes - such as being male, being younger in age, being married, and having a lower body mass index - showed a favorable association with quality of life. Strict interventions and preventative measures are essential for addressing musculoskeletal issues in the neck and shoulder region, as indicated by these findings. Further research should be dedicated to developing tailored interventions that specifically target different demographic cohorts, with the ultimate goal of improving the quality of life for all.

## Introduction

Musculoskeletal symptoms are a significant factor leading to primary healthcare visits [1]. Musculoskeletal pain refers to pain that affects different structures of the musculoskeletal system, such as muscles, bones, tendons, ligaments, and nerves [2]. Among the different types of musculoskeletal complaints, neck and shoulder pain (NSP) is recognized as a common complaint that can lead to disability in adults. Due to the inadequate localization of pain in this area, NSP is frequently treated and managed as a singular entity. Neck and shoulder pain has been linked to particular physical activities, including occupational characteristics, computer use, posture, and psychosocial factors [3].

Previous research conducted in Sweden and Norway examined the occurrence of NSP, revealing a one-year prevalence rate of 26% and 34%, respectively [4-6]. A further study conducted in Sweden found that the prevalence of NSP was 18% on average [6,7]. The occurrence of lower back pain is more common than NSP; yet, NSP has a notable influence on disability and the overall quality of life [8]. Lower back pain and NSP are the primary reasons for work-related unplanned absences and early retirements, accounting for the majority of musculoskeletal complaints [9]. According to data from the insurance industry, 18% of disability payments for musculoskeletal illnesses were attributed to problems related to the neck and shoulder [8]. Neck and shoulder pain carries a psychological and social burden, affecting both physical well-being and quality of life. It is linked to social and psychological stress and has a detrimental impact on patients' financial situation and overall quality of life [8,10]. A number of studies were conducted to investigate the occurrence and effects of NSP on dentists in Saudi Arabia [11-14]. Nevertheless, there remains a dearth of data concerning the influence of NSP on the overall population. The prevalence of NSP in Saudi Arabia was estimated to range between 38.2% and 53.5% for neck pain and between 21.6% and 34.4% for shoulder pain [15,16]. This study aimed to evaluate the impact of NSP on the health-related quality of life (HRQoL) of adult persons residing in Saudi Arabia and to identify its associated predictors.

## Materials and methods

Study design and population

This is a cross-sectional study using an online-administered questionnaire that was distributed via online platforms in Saudi Arabia for a duration between January and June 2023. All individuals older than 18 years living in Saudi Arabia and willing to participate in the study formed the study population. Participants who were non-communicative and had an intellectual disability were excluded from the study.

Participants recruitment

Participants were recruited using a convenient sampling technique. The study link was distributed through social media platforms (Facebook, WhatsApp, Instagram, and Twitter). This sampling method has a number of inherent advantages, including being time-efficient, economical, and straightforward to implement.

Study instrument

Participants will be required to fill out a 51-question survey, anonymously. Questions are grouped into five groups, each containing multiple questions covering the following aspects: informed consent, demographic data (gender, age category, nationality, marital status, education level, occupation, monthly income, area of residency, smoking status, and body mass index {BMI}), NSP profile (dominant hand, whether they have had any history of trauma, surgery, or congenital deformities in their neck and shoulders area, and whether they had muscle tension, stiffness, pressure, or dull pain in neck and shoulders area), and quality of life. Neck and shoulder pain refers to any sensations of discomfort, rigidity, or pain that occur in the area of the neck or shoulder. This pain can be categorized as either acute or chronic and can range in severity from mild to severe. Health-related quality of life pertains to an individual's subjective assessment of their physical and mental well-being across a period of time. It includes several components of overall health, such as physical, mental, emotional, and social functioning. The SF-36 questionnaire was used to evaluate HRQoL in our study.

The 36-Item Short Form Health Survey (SF-36) questionnaire was used to estimate participants' quality of life (QoL). The SF-36 questionnaire is widely utilized as an assessment tool for evaluating health-related quality of life. The SF-36 assesses eight dimensions: physical functioning (PF), role physical (RP), bodily pain (BP), general health (GH), vitality (VT), social functioning (SF), role emotional (RE), and mental health (MH) [17].

Sample size

The requisite minimal sample size is 170, ascertained by applying the subsequent formula and supposing an 87.4% quality of life of NSP in previous literature [10].

n= (Z^2^×p×{1−p})/e^2^

Here, n is required sample size, Z is z score corresponding to the 95% confidence level (which is equal to 1.96), p is estimated quality of life of NSP in the population, and e is margin of error (which is equal to 0.05).

Statistical analysis

Data were analyzed via SPSS software version 29 (Armonk, NY: IBM Corp.). Categorical variables were presented using frequency and percentage. Continuous variables were presented using mean and SD for normally distributed variables and median (interquartile range {IQR}) for non-normally distributed variables. Binary logistic regression analysis was used to identify predictors of better QoL using the mean SF-36 score (which is equal to 58.2) as the cut-off point for the dummy variable in the regression model). The level of significance was assigned as 5%.

## Results

Table 1 below presents the demographic characteristics of the study participants. A total of 6601 participants were involved in this study. More than half of them (72.3%, n = 4340) were females and aged 18-29 years (53.6%, n = 3214). The vast majority of them (90.1%, n = 5407) were Saudis. Around 52.8% (n = 3167) of the participants were single. Around half of the participants (54.3%, n = 3257) reported that they hold a bachelor’s degree. Around one-third (31.6%, n = 1895) of the participants were students. Around 54.1% (n = 3248) of the participants reported that their monthly income is less than 5000 Saudi Arabian riyal (SAR). Around 27.4% (n = 1645) of them reported that they live in the central area. Around 19.9% (n = 1313) of the participants reported that they were smokers. The median BMI for the study participants was 25.3 kg/cm^2^ (IQR: 21.5-29.6).

**Table 1:** Participants' demographic characteristics.

Variable	Frequency	Percentage
Gender		
Female	4340	72.3%
Age category		
18-29 years	3214	53.6%
30-39 years	918	15.3%
40-49 years	1048	17.5%
50-59 years	635	10.6%
60-60 years	152	2.5%
70-79 years	24	0.4%
80 years and above	10	0.2%
Nationality		
Saudi	5407	90.1%
Marital status		
Single	3167	52.8%
Married	2572	42.9%
Divorced	175	2.9%
Widowed	87	1.4%
Education level		
Primary school or lower	129	3.6%
High school	1611	26.8%
Diploma	586	9.8%
Bachelor’s degree	3257	54.3%
Higher education	328	5.5%
Occupation		
Student	1895	31.6%
Education sector	941	15.7%
Office work	735	12.2%
Healthcare provider	551	9.2%
Fieldwork	240	4.0%
Freelancer	245	4.1%
Industry and craft labor	84	1.4%
Unemployed	1310	21.8%
Monthly income		
Less than 5000 SAR	3248	54.1%
5000-10000 SAR	1118	18.6%
10000-15000 SAR	786	13.1%
15000-20000 SAR	526	8.8%
More than 20000 SAR	323	5.4%
Area of residency		
Central area	1645	27.4%
Northern area	1119	18.6%
Southern area	490	8.2%
Eastern area	1521	25.3%
Western area	1226	20.4%
Smoking status		
Sometimes	442	7.4%
Yes, for more than five years	337	5.6%
Usually	277	4.6%
Often	201	3.3%
No	4744	79.1%
Body mass index (BMI) (median {interquartile range})	25.3 (21.5-29.6) kg/cm^2^	

Participants’ neck and shoulder pain profile

Table 2 below presents the pain profile of the study participants. The majority of the study participants (86.6%, n = 5196) reported that their dominant hand was the right hand. Around one-quarter of the participants (27.2%, n = 1634) reported that they have a history of trauma, surgery, or congenital deformities in the neck and shoulder area. The majority of the participants (76.8%, n = 4610) reported that they had muscle tension, stiffness, pressure, or dull pain in the neck and shoulder area. The mean pain score for the study participants was 4.0 (SD: 2.9), which indicates mild degree of pain. The majority of the study participants (76.3%, n = 4581) reported that their pain stayed for less than six weeks. When the participants were asked about their health today, the mean score was 5.9 (SD: 3.9), which indicates moderate health status.

**Table 2:** Participants' pain profile.

Variable	Frequency	Percentage
Dominant hand		
Right hand	5196	86.6%
Left hand	475	7.9%
Both hands	330	5.5%
Have you had any history of trauma, surgery, or congenital deformities in your neck and shoulder area?		
Yes	1634	27.2%
Have you ever had muscle tension, stiffness, pressure, or dull pain in the neck and shoulder area?		
Yes	4610	76.8%
Mean pain score (between 0, representing “no pain,” and 10, representing “the worst pain imaginable”)	4.0 (2.9)	
For how long do you have this pain		
Less than 6 weeks	4581	76.3%
Between 6 weeks and 6 months	497	8.3%
More than 6 weeks	923	15.4%
Please indicate on the scale how your health is today (the best health state you can imagine is marked 0 and the worst health state you can imagine is marked 10) (mean score {SD})	5.9 (3.9)	

Predictors of better quality of life

The mean SF-36 score for the study participants was 58.16 (17.4), which demonstrates moderate quality of life. Binary logistic regression analysis showed that male gender, younger age, being married or divorced, being educated, and having lower BMI (less than 25.3 kg/cm^2^) were predictors of better QoL (p < 0.05) (Table 3). The adjusted regression analysis model controlling for age, gender, occupation, smoking status, and BMI showed that being educated is a predictor of having better QoL (p < 0.05). On the other hand, higher income level was associated with lower QoL (p < 0.05).

**Table 3:** Binary logistic regression analysis. *P<0.001 was considered significant. **P<0.05 was considered significant. ***P<0.01 was considered significant. ****Adjusted odds ratio taking into consideration the following confounders “age, gender, occupation, smoking status, and BMI.” This table presents the odds ratio of having a better quality of life.

Variable	Odds ratio of having better QoL using SF-36 score (95% confidence interval)	Adjusted odds ratio of having better QoL using SF-36 score (95% confidence interval)****
Gender		
Female (reference category)	1.00	-
Male	1.26 (1.13-1.41)*	
Age category		
18-29 years (reference category)	1.00	-
30-39 years	0.94 (0.81-1.09)	
40-49 years	0.54 (0.47-0.63)*	
50-59 years	0.63 (0.53-0.75)*	
60-60 years	0.51 (0.36-0.71)*	
70-79 years	0.16 (0.06-0.48)*	
80 years and above	-	
Nationality		
Non-Saudi (reference category)	1.00	1.00
Saudi	1.15 (0.97-1.37)	1.16 (0.98-1.40)
Marital status		
Widowed (reference category)	1.00	1.00
Single	0.99 (0.58-1.68)	1.03 (0.63-1.68)
Married	2.00 (1.46-2.73)*	1.17 (0.73-1.87)
Divorced	1.48 (1.08-2.03)**	0.89 (0.51-1.56)
Education level		
Higher education (reference category)	1.00	1.00
Primary school or lower	7.71 (2.65-22.41)*	4.79 (1.60-14.39)*
High school	6.80 (2.38-19.43)*	3.60 (1.23-10.60)**
Diploma	5.94 (2.06-17.12)*	3.68 (1.24-10.92)**
Bachelor’s degree	7.95 (2.79-22.65)*	4.68 (1.59-13.76)***
Occupation		
Industry and craft labour (reference category)	1.00	-
Fieldwork	0.77 (0.58-1.01)	
Student	0.92 (0.76-1.11)	
Education sector	0.51 (0.41-0.63)*	
Freelancer	0.58 (0.43-0.79)*	
Office work	0.72 (0.58-0.90)***	
Healthcare provider	0.67 (0.55-0.82)***	
Monthly income		
Less than 5000 SAR (reference category)	1.00	1.00
5000-10000 SAR	0.75 (0.58-0.98)*	0.84 (0.64-1.11)
10000-15000 SAR	0.89 (0.67-1.18)	1.01 (0.75-1.36)
15000-20000 SAR	0.72 (0.56-0.93)*	0.72 (0.55-0.94)*
More than 20000 SAR	0.87 (0.69-1.09)	0.66 (0.51-0.86)**
Smoking status		
Sometimes (reference category)	1.00	-
Yes, for more than five years	0.83 (0.68-1.09)	
Usually	0.80 (0.64-0.99)*	
Often	0.67 (0.53-0.86)**	
No	0.58 (0.43-0.77)***	
Body mass index category		
Less than 25.3 kg/cm^2 ^(reference category)	1.00	-
25.3 kg/cm^2^ and above	0.71 (0.64-0.79)***	

Table 4 below presents the distribution of study participants based on their NSP stratified by their QoL score. There was a statistically significant difference in the proportion of study participants based on their NSP and their QoL score (p<0.001).

**Table 4:** The distribution of participants based on their NSP stratified by their QoL. NSP: neck and shoulder pain; QoL: quality of life

Variables	Higher QoL	Lower QOL	p-Value
With NSP	2173 (47.1%)	2437 (52.9%)	<0.001
Without NSP	837 (60.2%)	554 (39.8%)	

## Discussion

Musculoskeletal pain is becoming increasingly prevalent and poses significant socioeconomic challenges that raise the necessity of creating tailored treatment plans and effective management approaches that address individual patient needs [18]. This study aimed to evaluate the impact of NSP on HRQoL of adult persons residing in Saudi Arabia and to identify its associated predictors.

The study results found that around one-quarter of the participants (27.2%) reported that they have a history of trauma, surgery, or congenital deformities in the neck and shoulder area. In our study, we found that 29.9% of the participants who reported NSP were found to have a history of trauma, surgery, or congenital deformities in the neck and shoulder area. Where it was found that neck pain and stiffness after trauma can be caused by congenital defects in the posterior arch of the atlas [19]. Also, NSP was found to be significantly present postoperative with patients with neck and shoulder surgery [20,21].

Additionally, the study results found that the majority of the participants (76.8%) reported that they had muscle tension, stiffness, pressure, or dull pain in neck and shoulder area. In fact, various factors can contribute to muscle tension, stiffness, and pain in the neck and shoulders, where it was found that delayed onset muscle soreness (DOMS) can lead to decreased pressure pain thresholds in these areas [22]. Besides, repetitive light work can lower the pressure pain threshold in the trapezius muscles, leading to increased discomfort [23]. Moreover, psychosocial factors were found to affect these symptoms as well [24-26].

In our study, when the participants were asked about their health today, the mean score was 5.9 (SD: 3.9), which indicates moderate health status. In addition, the mean pain score for the study participants was 4.0, which indicates mild degree of pain, where this indicates that patients do not experience sever issues with their necks and shoulders. However, other studies revealed a significantly increased prevalence of neck and shoulder pain in Saudi Arabia, particularly among specific occupational groups. Construction workers, healthcare professionals, and medical students have all been found to experience significant levels of musculoskeletal pain in these areas [27-30]. Although, the study results found that the mean SF-36 score for the study participants was 58.16, which demonstrates moderate quality of life. In addition, the majority of the study participants (76.3%) reported that their pain lasted for less than six weeks, and it's important to note that a longer duration of complaints can predict poorer outcomes for patients with neck and shoulder pain [31]. Where this increased quality of life and shorter pain duration mostly resulted from proper management provided for the patients, effective management is crucial. Physiotherapy, including exercise and mobilization, has been identified as an effective treatment option [32]. Furthermore, active exercises, particularly those emphasizing proprioceptive training, have been found to be more effective than passive physiotherapy [33]. Additionally, a neck and shoulder stretching exercise program has been shown to significantly decrease pain and improve function and quality of life [34].

Furthermore, in our study, binary logistic regression analysis showed that male gender, younger age, being married or divorced, and having lower BMI (less than 25.3 kg/cm^2^) were predictors of better QoL. Indeed, older age, female gender, and weight are risk factors for musculoskeletal pain [13,35]. A notable correlation was found between age, gender, and the prevalence rate of neck and shoulder pain [36]. Additionally, lack of physical activity and elevated BMI are linked to higher chances of experiencing chronic neck and shoulder pain among adults in the general population [37].

Patient-customized treatment plans are essential for addressing musculoskeletal pain, especially in the neck and shoulders, in Saudi Arabia. Factors like trauma, surgery, and psychosocial issues contribute to this pain. Effective management strategies, including physiotherapy and exercises like stretching, can significantly improve quality of life. Understanding predictors of better quality of life, like male gender, younger age, and lower BMI, is crucial for interventions. It's important to address risk factors, such as older age, lack of physical activity, and high BMI to prevent chronic pain. Implementing these strategies can reduce the socioeconomic burden of musculoskeletal pain and enhance well-being in Saudi Arabia.

The present study is not without limitations. Because of the cross-sectional design, it was not possible to establish a causal relationship between the variables under investigation. Potentially, the implementation of an online survey could have limited the applicability of the results of our research. Our findings should therefore be interpreted with caution.

## Conclusions

Despite the participants' moderate assessments of their present health and quality of life, specific demographic characteristics - including male gender, younger age, marital status, and reduced BMI - exhibited a positive association with quality of life. In order to address musculoskeletal issues in the neck and shoulder region, targeted interventions and preventative measures are crucial according to these results. Additional investigation should be devoted to customized interventions targeting distinct demographic groups in order to enhance the overall quality of life.
